# Biocompatibility of a Magnetic Tunnel Junction Sensor Array for the Detection of Neuronal Signals in Culture

**DOI:** 10.3389/fnins.2018.00909

**Published:** 2018-12-12

**Authors:** Daniela Moretti, Mattia Lorenzo DiFrancesco, Parikshit Pratim Sharma, Silvia Dante, Edoardo Albisetti, Marco Monticelli, Riccardo Bertacco, Daniela Petti, Pietro Baldelli, Fabio Benfenati

**Affiliations:** ^1^Center of Synaptic Neuroscience and Technology, Istituto Italiano di Tecnologia, Genova, Italy; ^2^IRCCS Ospedale Policlinico San Martino, Genova, Italy; ^3^Department of Physics, Politecnico di Milano, Milan, Italy; ^4^Department of Nanoscopy & NIC@IIT, Istituto Italiano di Tecnologia, Genova, Italy; ^5^IFN-CNR, Politecnico di Milano, Milan, Italy; ^6^Department of Experimental Medicine, University of Genova, Genova, Italy

**Keywords:** magnetic tunnel junction (MTJ), biocompatibility, neuron culture, bio-magnetic field, *in vitro*, sensor

## Abstract

Magnetoencephalography has been established nowadays as a crucial *in vivo* technique for clinical and diagnostic applications due to its unprecedented spatial and temporal resolution and its non-invasive methods. However, the innate nature of the biomagnetic signals derived from active biological tissue is still largely unknown. One alternative possibility for *in vitro* analysis is the use of magnetic sensor arrays based on Magnetoresistance. However, these sensors have never been used to perform long-term *in vitro* studies mainly due to critical biocompatibility issues with neurons in culture. In this study, we present the first biomagnetic chip based on magnetic tunnel junction (MTJ) technology for cell culture studies and show the biocompatibility of these sensors. We obtained a full biocompatibility of the system through the planarization of the sensors and the use of a three-layer capping of SiO_2_/Si_3_N_4_/SiO_2_. We grew primary neurons up to 20 days on the top of our devices and obtained proper functionality and viability of the overlying neuronal networks. At the same time, MTJ sensors kept their performances unchanged for several weeks in contact with neurons and neuronal medium. These results pave the way to the development of high performing biomagnetic sensing technology for the electrophysiology of *in vitro* systems, in analogy with Multi Electrode Arrays.

## Introduction

In the last 20 years, the study of the magnetic field generated by the electrical activity of the brain has revolutionized neuroscience. In particular, magnetoencephalography (MEG), due to its unprecedented spatial and temporal resolution (Sander et al., 2012; Borna et al., 2017), gained wide clinical applications in detecting and localizing pathological cortical activity in patients with brain tumors or intractable epilepsy (Stufflebeam, 2011). Despite the wide use of this new technique, the nature of MEG signals at local level is still not completely understood (Haueisen and Knösche, 2012). Furthermore, during the last decade, there has been an increasing need to extend biomagnetic signal detection to microscale for higher spatial resolution and system integration in real-time and robust processes (Shen et al., 2018). The big challenges to overcome are (i) preserving the weak biomagnetic signals that can be easily polluted by environmental noise and (ii) detecting magnetic signals, which are heavily damped even at short distances. As described in (Hall et al., 2012), the magnetic field decays one order of magnitude faster than the electric field. In this context, there is evident crucial interest in developing devices for local magnetic recording in *in-vitro* systems.

Growing primary neuronal cultures directly on top of magnetic sensors offers the possibility to minimize the distance between the source of the biological signal and the detector to maximize sensitivity. However, this brings up potential issues related to the biocompatibility of magnetic sensors and to the viability of neurons in direct contact with them. In addition, in order to maximize the sensitivity to the magnetic signal, one has to take into account that the magnetic field associated with the propagation of the action potential along the axon arises mainly from the axial intracellular currents and is directed perpendicular to the axon (Roth and Wikswo, 1985; Hall et al., 2012).

In the field of magnetic sensors for *in-vitro* applications, attempts (Barry et al., 2016) have been made by using the nitrogen-vacancy quantum defects in diamond to detect the magnetic field produced by action potentials in the squid and worm giant axons. Using a different technology, promising candidates for the detection of the magnetic field *in-vitro* are magnetoresistive sensors (Graham et al., 2004) based on Giant Magnetoresistance (GMR) (Martins et al., 2009; Gaster et al., 2011) or Tunneling Magnetoresistance (TMR) (Albisetti et al., 2013; Sharma et al., 2017a) due to their high sensitivity, electrical readout, capability to work at room temperature (RT) and potential compatibility with a cell culture.

Due to the long-term biocompatibility requirements and the high sensitivity of cultured neurons to toxic elements (in particular metals) that can be released by the devices immersed in a saline solution, no attempts have been carried out so far for primary neuronal cultures. Recent studies assessing the capability of these magnetoresistive sensors in detecting a magnetic field of biological origin at the local scale (Barbieri et al., 2016; Caruso et al., 2017) concern macroscopic *in vitro* and *in vivo* systems, such as muscle or visual cortex, except for Sharma et al. (2017b), in which very preliminary results on cell viability were shown for neurons cultured for 2 weeks on magnetic tunneling junctions (MTJ) protected by 170 nm of capping layers.

However, a detailed study of the biocompatibility of the system aimed at the optimization of the sensitivity of the platform is still missing. As previously mentioned, low thicknesses of the capping layers are required to overcome the fast decay of the magnetic field. In addition, one has also to take into account that magnetoresistive sensors are sensitive to external magnetic field along only one axis, depending on the magnetic anisotropies of their reference magnetic layers (Sharma et al., 2016).

In the present work we investigate in detail the biocompatibility of MTJ sensors with murine embryonic hippocampal neurons cultured on the top of the device by viability assays, immunocytochemistry and patch-clamp recordings. We studied the dendritic and axonal growth, the formation of synaptic connections and the maturation of the firing properties. Moreover, engineered cultures are grown on top of the sensors in order to maximize the sensitivity of the neuronal-sensor interface. We show that our devices are fully biocompatible up to 3 weeks and preserve their physical integrity and performance.

## Materials and Methods

Magnetic tunneling junction-based sensors are composed of a sensor stack that contains several materials, as explained in detail in the following paragraph. A potentially issue in terms of biocompatibility is the presence of neurotoxic materials in the sensor stack, such as cobalt or manganese. Even if these materials are present in low quantity and are isolated by capping layers, we first studied their potential noxious effects on neuronal growth and survival by using *ad hoc* samples (CoFe films) that mimic the worst case with respect to Magnetic tunneling junction-based sensors.

### Fabrication of MTJs and CoFe Films

**Figure 1:**
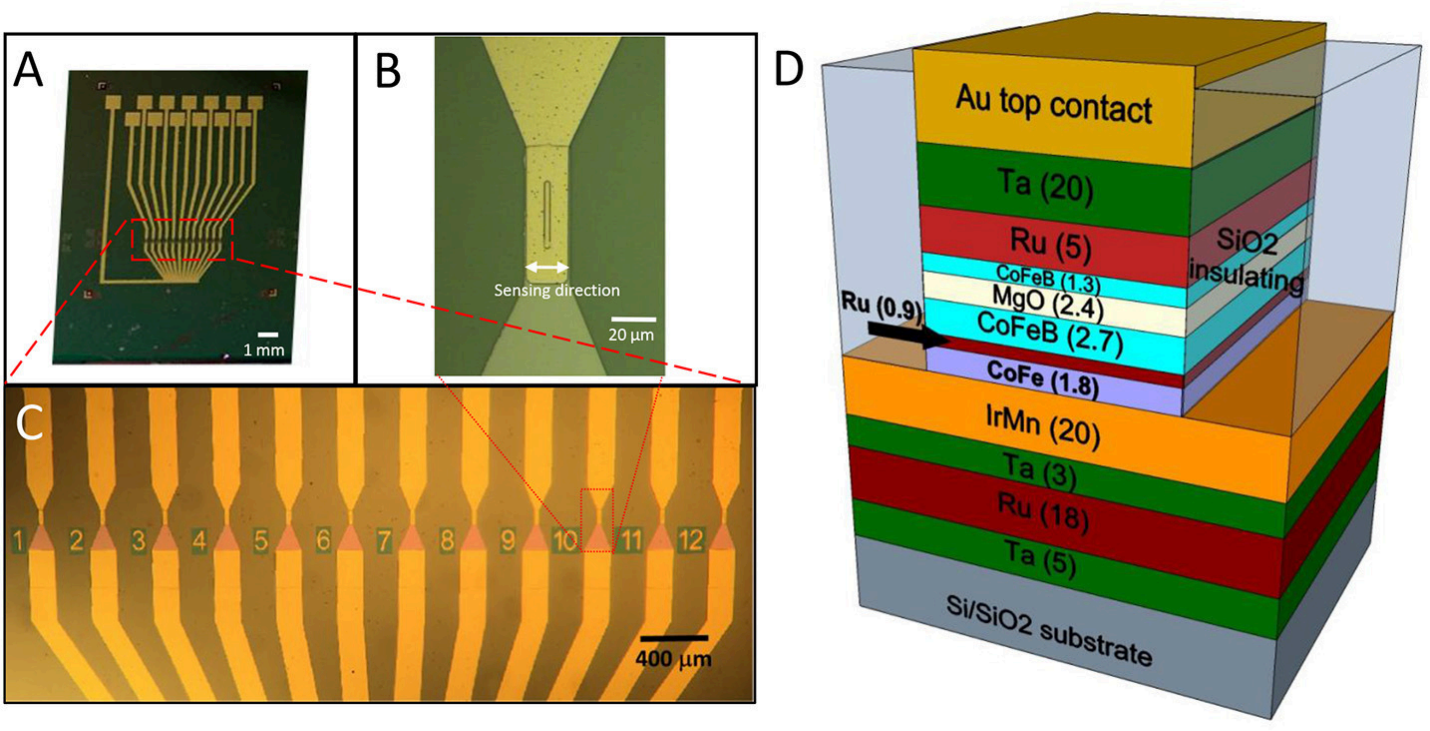
**(A)**Optical image of the chip featuring the 12 sensors, each provided with an independent contact and a common ground contact; **(B)** Zoom image of a single sensor area (4 × 30 μm ^2^); **(C)** Zoom image of the sensor active region, composed of 12 magnetic tunneling junctions; **(D)** Structure of a magnetic tunneling junction (thickness of the layers in nm).

Magnetic tunneling junction-based sensors were grown on Si/SiO_2_ substrates by magnetron sputtering with a base pressure of 2·10^−9^ Torr and an applied magnetic field of 300 Oe (Albisetti et al., 2013, 2014). The multilayer is composed by (thickness in nm): Ta(5)/ Ru(18)/ Ta(3)/ Ir_20_Mn_80_(20)/ Co_60_Fe_40_(1.8)/ Ru(0.9)/Co_40_Fe_40_B_20_(2.7)/ MgO(2.5)/ Co_40_Fe_40_B_20_(1.3)/ Ru(5)/Ta(20). Co_60_Fe_40_ and MgO layers were deposited in RF mode while the remaining layers were grown in DC mode. After deposition, the samples were processed with optical lithography and ion beam etching to obtain sensor chips featuring 12 MTJ sensors arrays (Figure 1). Each MTJ sensor has a 3 × 40 μm^2^ junction area (Figure 1B). A 110 nm thick SiO_2_ layer was deposited for insulating purposes by magnetron sputtering. Afterwards, a Ti(7)/ Au(100) bilayer was deposited by magnetron sputtering for the contacts. Each sensor is provided with two contacts, the top one to address independently every device, and the bottom one connected to the common ground. An additional step of lithography was performed to planarize the devices, since the two ion milling processes used for the tunnel junction definition and the subsequent contacts deposition leave the sidewalls of the top layers of the magnetic junctions exposed. If the step height between the device top contact and the SiO_2_ surrounding is higher than, or comparable with, the thickness of the capping layers, the sidewalls of the junctions cannot be completely sealed. This could imply problems with the biocompatibility and the endurance of the sensors. The planarization of the device was obtained through liftoff, by depositing 180 nm SiO_2_ by magnetron sputtering around the devices, giving rise to a step of about 30 nm between the top layer and the substrate. The sensors arrays were then annealed at 310°C at a pressure of 10^−6^ Torr for 1 h; this step was performed to enable the crystallization of the ferromagnetic layers and the tunnel barrier, with the aim of increasing the performance of the sensor (Sharma et al., 2016). In this case, sensitivities in the nT range can be easily achieved (Almeida et al., 2008; Sharma et al., 2017b), while pT sensitivities can be obtained with different strategies. Structures with top-pinned sensing layer and bottom-pinned reference layer present a linear response arising from the cross anisotropies of the two electrodes and up to 60 pT/Hz^∧^0.5 of sensitivity (Ferreira et al., 2012; Sharma et al., 2016). In addition, a combination of magnetorestrictive and magnetoresistive effects can theoretically enhance the sensitivity of magnetic tunneling junctions up to fT (Pertsev, 2016). Finally, another approach is the use of flux guides, which can concentrate and enhance the magnetic signal in correspondence of the sensor (Chaves et al., 2008).

As last step of the fabrication of our sensors, a capping layer composed by a three-layer of (thickness in nm): SiO_2_(50)/Si_3_N_4_(25)/SiO_2_(50) was deposited. In this case, the SiO_2_ layer was deposited by e-beam evaporation (E-beam Evaporator Evatec BAK 640), while Si_3_N_4_ was grown by magnetron sputtering. The first SiO_2_ layer is used as adhesive layer, while Si_3_N_4_ film protects the sensor from the liquid environment, due to its low porosity and high chemical resistance (Vanhove et al., 2013). The final layer of SiO_2_ is used to promote the adhesion of the extracellular matrix proteins used for the cell growth (Monticelli et al., 2016).

Cobalt Iron (Co_60_Fe_40_) films, 20 nm thick, were grown on Si/SiO_2_ substrates by magnetron sputtering in an AJA Orion8 system with a base pressure of 2·10^−9^ Torr. Since Cobalt Iron is the most toxic material among those used for MTJs, these samples represent the hardest condition to achieve a neuronal growth. After the deposition, the same capping layer used for MTJs, made of SiO_2_ (50 nm)/Si_3_N_4_ (25 nm)/SiO_2_(50 nm), was deposited as discussed above.

### Preparation of MTJ Chip and CoFe Films for Cell Culture

Samples were first sterilized by immersion in ethanol washed twice in sterile water, dried in a laminar flow hood and further sterilized by UV irradiation for 1 h. The day before dissection substrates were coated with 0.05–0.1% polyethyleneimine (PEI, MW 25.000, Sigma-Aldrich) solution for 45 min. Then, the PEI solution was removed and substrates were washed 4 times with sterile distilled water and stored at 4°C. To perform random neuronal growth on the samples, the day of dissection, substrates were coated with 100 μl laminin (Sigma-Aldrich) [20 μg/ml] and incubated at 37°C for 2 h. Laminin was removed by aspiration prior to seeding dissociated neurons. All biosecurity and safety procedures were followed, as specifically required by the Health & Safety Office of the Istituto Italiano di Tecnologia (Genova, Italy).

### Engineered Cultures on MTJ

Since the weak biomagnetic signals can be easily polluted by environmental noise, it is essential to reduce the distance between the sensor and biological source. To overcome this issue, we propose a platform to detect neuronal activity in cell cultures, where neurons are grown on top of the magnetic sensor and the distance between the sensor and biological source is dramatically reduced. Moreover, since the magnetic sensors have one defined sensing direction (Figure 1B), a critical aspect is to maximize the sensitivity of the system by properly aligning the network to the devices. Considering that magnetic fields produced by the neuronal currents are forcing perpendicular to the current direction, in the most favorable configuration the axons have to be perpendicular to the sensing direction of the probe (Hall et al., 2012). For this reason, beside a condition of random neuronal growth, we also carried out controlled-topology neuronal networks, by neuronal processes to grow along the sensor's major axis.

To this aim, we added the following additional process to sample preparation. After the sterilization and PEI coating, MTJ chips were prepared for patterned coating deposition. Agarose (0.15% w/w in water; Sigma-Aldrich) was prepared by dissolving the agarose powder in MilliQ-water brought to its boiling point in a microwave oven for 2 min. Then, agarose (0.1 μl) was deposited on the sensor area and immediately aspirated to have a thin layer to inhibit random cellular adhesion. To obtain a neuronal network along the sensors, lines of PEI (80 × 20 μm) were deposited on the top of the agarose-coated 12-MTJ sensors by a nano-drop ink jet print (NanoEnabler, BioForce Nanoscience Inc.), equipped with SPT-C30 S cantilevers (NanoandMore), (Video S1). Relative humidity in the chamber was kept at 70 ± 5%. A small volume (0.3 μl) of the PEI solution was loaded into the cantilever reservoir by a micropipette. Glycerol (5% w/w in water; Sigma-Aldrich) was added to the solution to avoid evaporation. Contact force and withdrawal distance were fine controlled (0.002 nN and 40 μm, respectively), in order to obtain the desired size of the PEI lines. The PEI lines were deposited exactly on top of the MTJ sensors, after micrometric alignment of the chip in correspondence with the cantilever. MTJ chips were stored at 4°C. To improve the neuronal adhesion in the area surrounding sensor active region, laminin was applied. No laminin was deposited on the top of the 12-MTJ sensor where PEI lines were made. On the day of dissection 80 μl laminin [20 μg/ml] were deposited outside the sensor active region by 10 μl drops and incubated at 37°C for 2 h. Laminin was removed by aspiration prior to seeding dissociated neurons.

### Primary Neuronal Cultures

All procedures were carried out in accordance with the guidelines established by the European Communities Council (Directive of November 24th, 1986) and approved by the National Council on Health and Animal Care (authorization ID 227, prot. 4127, 25th March 2008). Primary hippocampal cultures were obtained from Sprague-Dawley rats at embryonic day 18 (E18) (Charles River). Pregnant females were deeply anesthetized with CO_2_ and decapitated. Embryos were removed and brains were placed in cold Hank's balanced salt solution (HBSS). After removal of the meninges, the hippocampus was carefully dissected, incubated with 0.125% trypsin for 15 min at 37°C and mechanically dissociated. Eighty thousands neurons were then plated on each coated devices. Cell culture were maintained in 2 ml of cell culture medium composed by: Neurobasal medium, 2% B-27, 1% penicillin–streptomycin, and 1% Glutamax and maintained at 37°C in 5% CO_2_.

### Viability Assay

Cells were incubated for 3 min at RT in extracellular medium (EM; NaCl 135 mM, KCl 5.4 mM, MgCl_2_ 1 mM, CaCl_2_ 1.8 mM, glucose 10 mM, Hepes 5 mM, pH 7.4), 5 mg/ml propidium iodide (PI), containing 15 μg/ml fluorescein diacetate (FDA) and 3.3 μg/ml Hoechst-33342. After incubation, cells were washed once in EM and immediately imaged. PI is a red-fluorescent nuclear and chromosome counterstain able to permeate exclusively through the membrane of dead cells, FDA a non-fluorescent molecule that is hydrolyzed to fluorescent fluorescein only within live cells and Hoechst a nuclear counterstain binding the DNA of both live and dead cells.

The hardware configuration for the imaging experiments was based on a Nikon Eclipse Ni-U upright microscope equipped with an epifluorescence attachment and a Camera DS-Qi2 (Nikon Instruments). Cells were magnified, with a 20x objective (0.75 NA). For each sample, at least 5 distinct fields of view were acquired. Considering the total number of nuclei identified by Hoechst fluorescence and the apoptotic nuclei, identified by PI fluorescence, the percentages of living cells were calculated for each field as: (Hoechst-positive nuclei – PI-positive nuclei) / (Hoechst-positive nuclei). FDA staining was used as a further marker of cell-membrane integrity and culture viability. Images were analyzed by using the Image J software. Statistical analysis was performed using a commercial package [Sigmastat, Systat Software Inc.].

### Immunocytochemical Analysis

Cells were washed twice in phosphate buffered saline (PBS) solution, fixed with 4% (w/v) paraformaldehyde in PBS at RT for 20 min and washed two times in PBS. Fixed samples were permeabilized with 0.1% (v/v) Triton X-100 in PBS for 5 min. Blocking solution (PBS, 1% BSA, 5% FBS) was added for 30 min at RT to block nonspecific reactions. The following primary antibodies, diluted in blocking solution, were used: monoclonal anti-MAP2 antibody (Synaptic Systems; mouse #188011, dilution 1:500), polyclonal anti-Tau (rabbit # 314002, dilution 1:1,000) and polyclonal anti-NeuN antibody (guinea pig, # 266004, dilution 1:500). MAP2 is the major microtubule associated protein of brain tissue. Tau is a microtubule-associated protein of the neuronal axons. NeuN is a neuron-specific DNA-binding protein present in most neuronal cell types. After incubation for 2 h at RT, samples were washed 3 times with PBS and incubated with fluorophore-conjugated secondary antibodies for 1 h at RT. Secondary antibodies were: anti-mouse Alexa Fluor 488 (#A11029; Thermo Fisher Scientific, dilution 1:1,000), anti-rabbit Alexa Fluor 647 (#A21245; dilution 1:1000) and anti-guinea pig Alexa Fluor 546 (#A11075; dilution 1:1,000). Samples were mounted using Mowiol 4-88 (81381, Sigma-Aldrich) and stored at 4°C. In the case of engineered cultures, samples were mounted using Prolong anti-fade reagent containing DAPI (a blue-fluorescent DNA stain, Invtrogen), without using anti-NeuN antibody. Confocal microscopy was performed using an SP8 microscope (Leica Microsystems GmbH) using 40x (1.3 NA) and 63x (1.4 NA) objectives. Confocal images were analyzed with the Leica LAS AF software (Leica Application Suite Advance Fluorescence, version 3.3, Leica Microsystems). Fluorescence microscopy was performed as described above.

### Electrophysiology

Whole-cell patch-clamp recordings of primary cortical rat neurons [14 and 21 days *in vitro* (DIV)] were performed using borosilicate glass patch pipettes (Kimble) pulled to a final resistance of 3–5 MΩ and under GΩ patch seal. Data were sampled at 20 kHz and low-pass filtered at 4 kHz with an EPC-10 Plus amplifier (HEKA Electronic). Recordings with leak currents >100 pA or series resistance >20MΩ were discarded. Data acquisition was performed using PatchMaster v2.73 software (HEKA Elektronic). Series resistance (Rs) was compensated 80% (2 μs response time) and the compensation was readjusted before stimulation. Potentials were not corrected for the measured liquid junction potential of 9 mV. All recordings were performed at 22–24°C. The extracellular “Tyrode” solution contained (in mM): 135 NaCl, 5.4 KCl, 1 MgCl_2_, 1.8 CaCl_2_, 5 HEPES, 10 glucose adjusted to pH 7.4 with NaOH, and the intracellular pipette solution contained (in mM): 126 K-Gluconate, 4 NaCl, 1 MgSO_4_, 0.02 CaCl_2_, 0.1 EGTA, 10 Glucose, 5 Hepes, 3 ATP-Na_2_, and 0.1 GTP-Na. Cell membrane capacitance was obtained from the slow time constant component used for capacitance compensation after reaching the whole-cell configuration, and Input resistance was calculated as the Current vs. Voltage (IV) curve slope measured at sub-threshold voltages. Measurements of the firing activity were performed in current-clamp configuration. Resting membrane potential (Vrest) was determined immediately after breakthrough in the whole-cell mode. Spontaneous firing activity was considered for analysis only from those cells with a Vrest between −70 and −50 mV, and the mean firing frequency was calculated as the average reciprocal of the interspike interval. Evoked firing activity was induced by injection of 5 pA current steps lasting 100 ms in neurons maintained at a holding potential (V_H_) of −70 mV through the injection of a negative current (I_H−70mV_). For each patched-neuron we calculated: the minimal current able to evoke the first Action Potential (current threshold), the maximum voltage reached at the Action Potential peak (AP peak), and the maximal rising slope (dV/dt) of the upstroke phase. Whole-cell currents were elicited in voltage-clamp configuration. A protocol consisting of a 200 ms voltage step from the holding potential of −70 to −100 mV, followed by 100-ms linear ramp up to 120 mV was used to evoke voltage-gated conductances. Sodium current peak was calculated as the minimum inward current value measured at the beginning of the ramp phase. Voltage-gated macroscopic currents were also evoked by stepping V_H_ from −60 to 20 mV for 30 ms with 10 mV increments with 2 s interpulse in order to quantify inward sodium currents (negative peak in the first 10 ms) and steady-state outward potassium currents in the last 5 ms. In all the protocols used, cells were clamped at a Vh of −70 mV before stimulation.

## Results

### MTJ Endurance

We first checked whether the magnetoresistive sensors were compatible with prolonged immersion in cell medium (basically an electrolytic solution of NaCl) at 37°C without deteriorating and loosing their sensor performances. Tunneling magnetoresistance measurements were performed using a Keithley 2611 source meter and an electromagnet driven by a Kepco power supplier. All the devices used here showed a resistance between 1 and 4 kΩ, which depends exponentially on the tunneling barrier thickness (Hayakawa et al., 2005).

**Figure 2:**
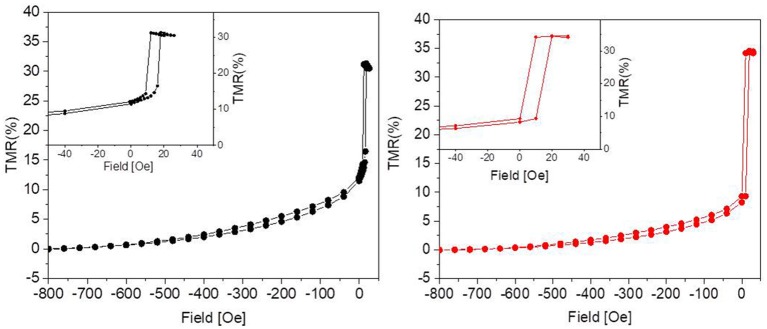
Percent TMR as a function of the applied magnetic field before **(Left)** and after **(Right)** 31 days in culture. The graphs show that the culture environment is not detrimental to the performance of the sensor, since the response to the external magnetic field remains unaltered. The insets show some residual hysteresis.

The tunneling magnetoresistance (TMR), defined as the normalized difference between the resistance for a given field and that in the parallel state of the magnetization in the two electrodes (i.e., for high negative fields in our case), is plotted in Figure 2. In the sensors used in this work, aiming at demonstrating the biocompatibility, maximum TMR variations between 20 and 50% were obtained, mainly depending on the CoFeB thickness used in the stack (Wiśniowski et al., 2008). A low-field sensitivity between 2 and 10 $\frac{\text{\%}}{\text{mT}}$ was achieved, together with a linear response and some residual hysteresis (see Figure 2, left, inset).

As reported in Figure 2, after 31 days in culture the devices presented TMR values comparable to the nominal ones, i.e., to those measured before the experiments. Since the tunneling is highly sensitive to any change in the device structure, we can therefore conclude that the capping layer is impermeable enough to prevent any interaction of the device with neurons and culture medium.

### Cell Viability on CoFe Film

**Figure 3:**
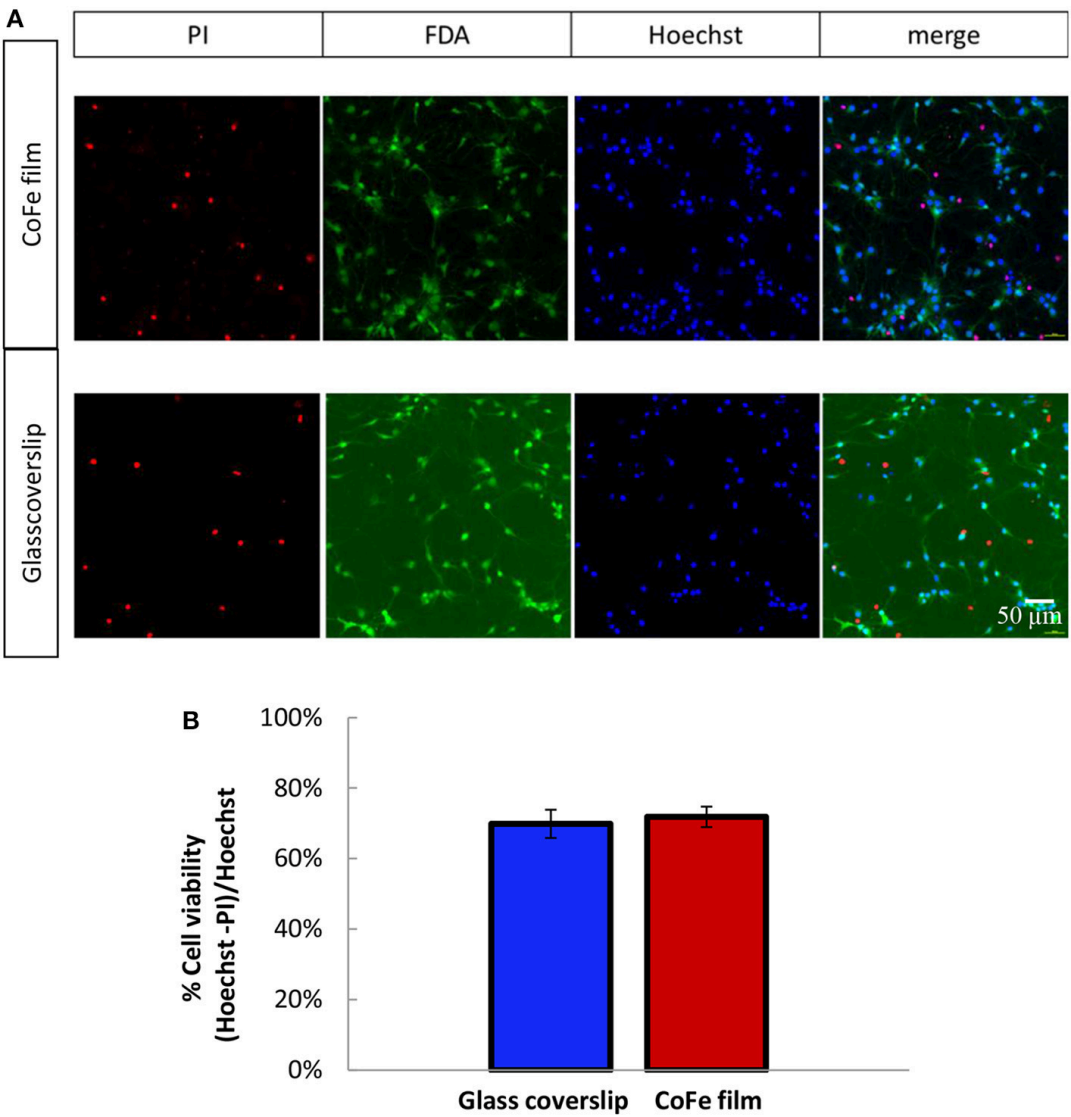
**(A)** Representative images of 11 DIV rat hippocampal neurons grown onto CoFe film (upper panels) or glass coverslips (lower panels) and stained with Hoechst (cell nuclei), fluorescein diacetate (FDA, live cells) and propidium iodide (PI, dead cells). **(B)** The bar histogram shows the means ± SE percentage of live cells (Hoechst and FDA positive) with respect to the total number of cell nuclei in cultures grown under control conditions (blue) or on CoFe films (red). Two-tailed unpaired Student's *t*-test. *p* = 0.52 (*n* = 6 per each condition, 3 independent preparations).

A crucial aspect for ensuring an efficient growth of a neuronal culture onto the sensor surface is to find a proper capping that enables cell viability in spite of the toxic materials of the sensor stack (i.e., cobalt) and, at the same time, provides protection to the magnetic device. To this aim, we studied cell viability on a substrate mimicking the situation of MTJs, but representing a worst case for cell viability in terms of thickness of toxic materials: a CoFe film 20 nm thick deposited on Si/SiO_2_ wafers and capped with a SiO_2_(50)/Si_3_N_4_(25)/SiO_2_(50) three-layer (thickness in nm). Cell growth displayed no qualitative differences between rat hippocampal neurons plated on the CoFe films or glass coverslips (Figure 3A). Quantitative analysis of cell viability was performed using a triple staining with PI, FDA and Hoechst as described in the Methods section

The microphotographs of panel (a) show that all the PI-positive cells were negative to FDA (dead cells), while all the PI-negative cell were also FDA-positive (live cells). The estimation of the percentage of live cells over the total cell nuclei showed that comparable cell viabilities, higher than 70%, were achieved for both neuronal populations grown on top of CoFe films or on top of control glass coverslips (Figure 3B).

We also achieved neuronal growth (data not shown) with CoFe films where the three-layer were grown by plasma enhanced chemical vapor deposition, instead of magnetron sputtering as described above. However, we dropped this technique because this three-layer did not sufficiently protect the CoFe layer due to infiltration issue. These findings validated the three-layer [SiO_2_ (50)/Si_3_N_4_ (25)/SiO_2_(50)] grown by magnetron sputtering that we chose for the CoFe films as suitable capping layers to isolate cells from the toxic materials of the CoFe films. For these reasons, we employed these capping layers to protect the MTJ sensor stack and to guarantee long-term biocompatibility.

### Proper Neuronal Growth on MTJ Chips

**Figure 4:**
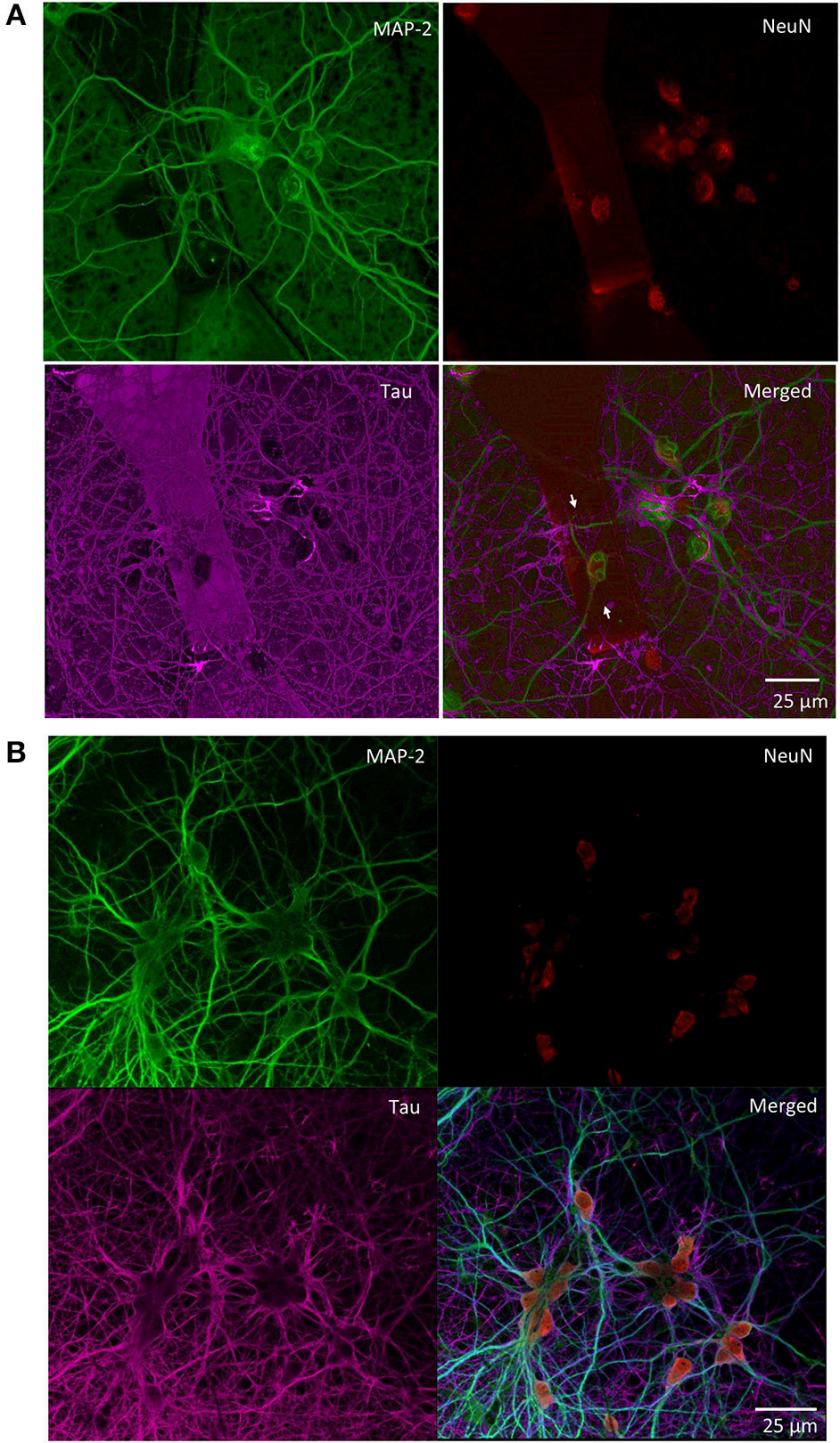
Primary rat hippocampal neurons grown with random topology at 14 DIV. Confocal images of the indicated immunoreactivities: MAP-2 in mature neuronal dendritic arborizations, NeuN in neuronal nuclei and Tau in axonal processes. **(A)** Neurons grown on the top of a MTJ sensor (white arrows indicate the border of the sensor area). **(B)** Neurons grown outside the sensor active region.

**Figure 5:**
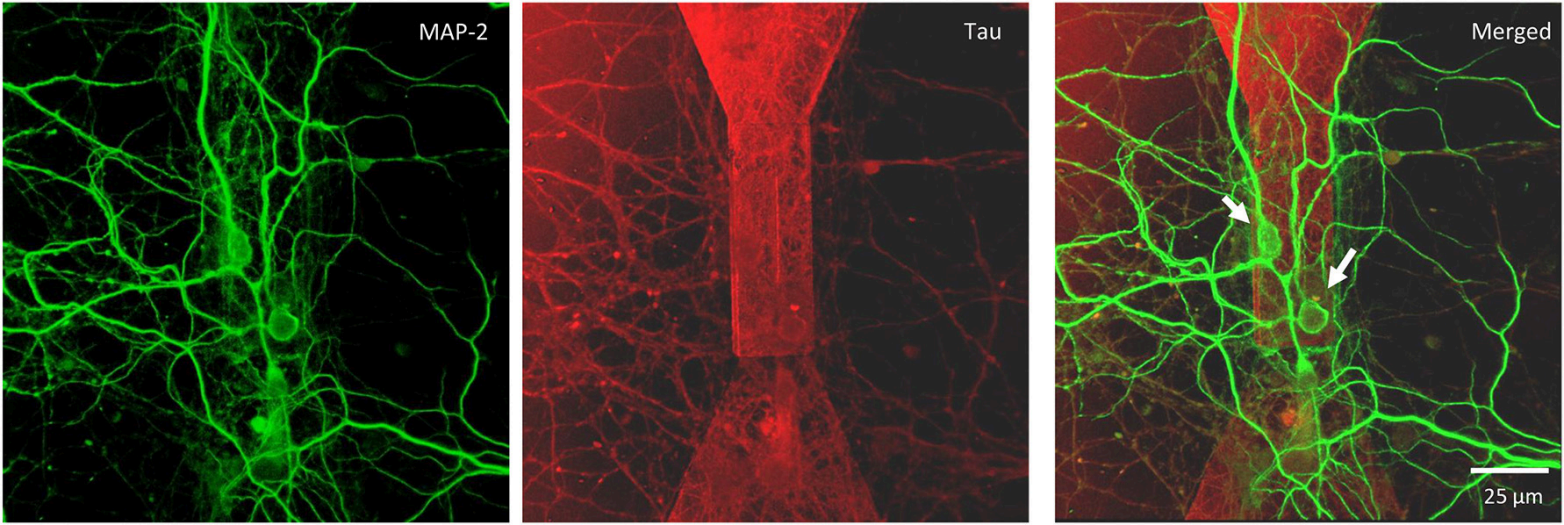
Confocal microscopy of random topology neuronal networks grown on top of a MTJ sensor. Rat hippocampal neurons at 14 DIV were stained with antibodies to MAP-2 (green) and Tau (red). White arrows indicate neurons on the top of the MTJ sensor.

We monitored the developing cultures up to 19 DIV by studying the expression of biomarkers of neuronal maturation including: (i) MAP-2 that is expressed only in neuronal cells and labels soma and dendrites, (ii) Tau that stains the axonal processes and (iii) NeuN that is a specific marker for neuronal nuclei. Figures 4A, 5 show a proper morphological differentiation of a rich neuronal network that homogenously developed on the top of the MTJ sensor. A similar picture was observed also in the periphery of the device (Figure 4B), where the immunostaining shows neurons with a correct growth of MAP-2 positive dendrites and tau-positive axons. Even in culture with random topology, the neuronal cell bodies adhered and developed correctly on the top of the MTJ junction. The device surface underneath the neuronal network is shown in the Tau microphotograph by the autofluorescence signal generated in this wavelength by the MTJ track materials (in particular metals). Moreover, in Figure 5, a couple of neurons (indicated with white arrows) spontaneously developed axons parallel to the MTJ. These observations lead us to conclude that no constraints were found on the whole chip surface for a correct neuronal culture growth. This result is important in order to show a proper adhesion and growth of delicate primary neurons on the top of the device and to rule out the potential noxious effect of the neurotoxic materials of the sensor stack (i.e., cobalt or manganese) and possibly of some residual traces of chemicals arising from the photoresist and solvents used in the lithographic process.

**Figure 6:**
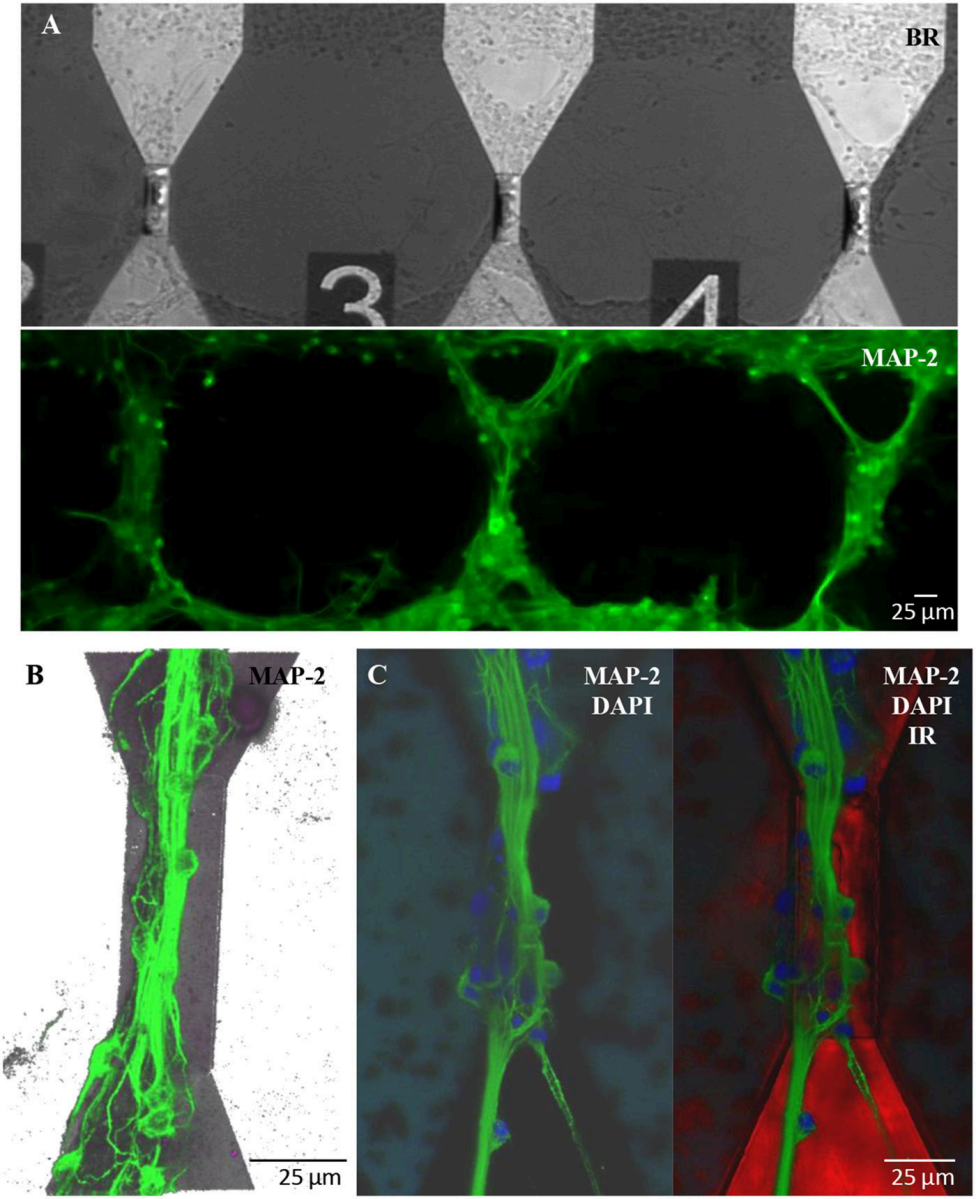
Engineered cultures of rat hippocampal neurons at 19 DIV. **(A)** Images obtained at the fluorescence microscope using bright field (above) and FITC filter for MAP-2- staining (green; lower panel). **(B)** Confocal images in a 3D reconstruction (z-stack: 35 μm) of neurons stained with MAP-2. **(C)** Confocal images stained with MAP2 (neuronal processes; green)+DAPI (nuclei; blue) (right) and merge image (left) with the sensor structure under the neuronal network (red; infrared exposure).

Similar results in terms of proper growth and development of the neuronal network were achieved in engineered cultures (Figure 6), where neurons were forced to grow with a controlled topology along the MTJ sensors to achieve the best system sensitivity. A reliable confinement of neuronal processes along the MTJ sensors was obtained over time (19 DIV). The PEI-patterned coating deposition enhanced cell adhesion along the MTJ sensors, while the background agarose layer successfully inhibited cell adhesion (Petrelli et al., 2013) outside the MTJ sensor tracks. Several cell nuclei (Figure 6C, blue) were located exactly on the top of the sensor and generated rich MAP-2-positive neurite bundles (Figures 6B,C, green) that were perfectly aligned along the sensor major axis, i.e., perpendicular to the MTJ sensing direction. This neuronal network architecture could achieve the best system sensitivity conditions: the distance between the source of the biological signal and the detector is minimized and the biological magnetic field is completely parallel to the sensing direction.

### Proper Spontaneous Electrical Activity on MTJ Chips

**Figure 7:**
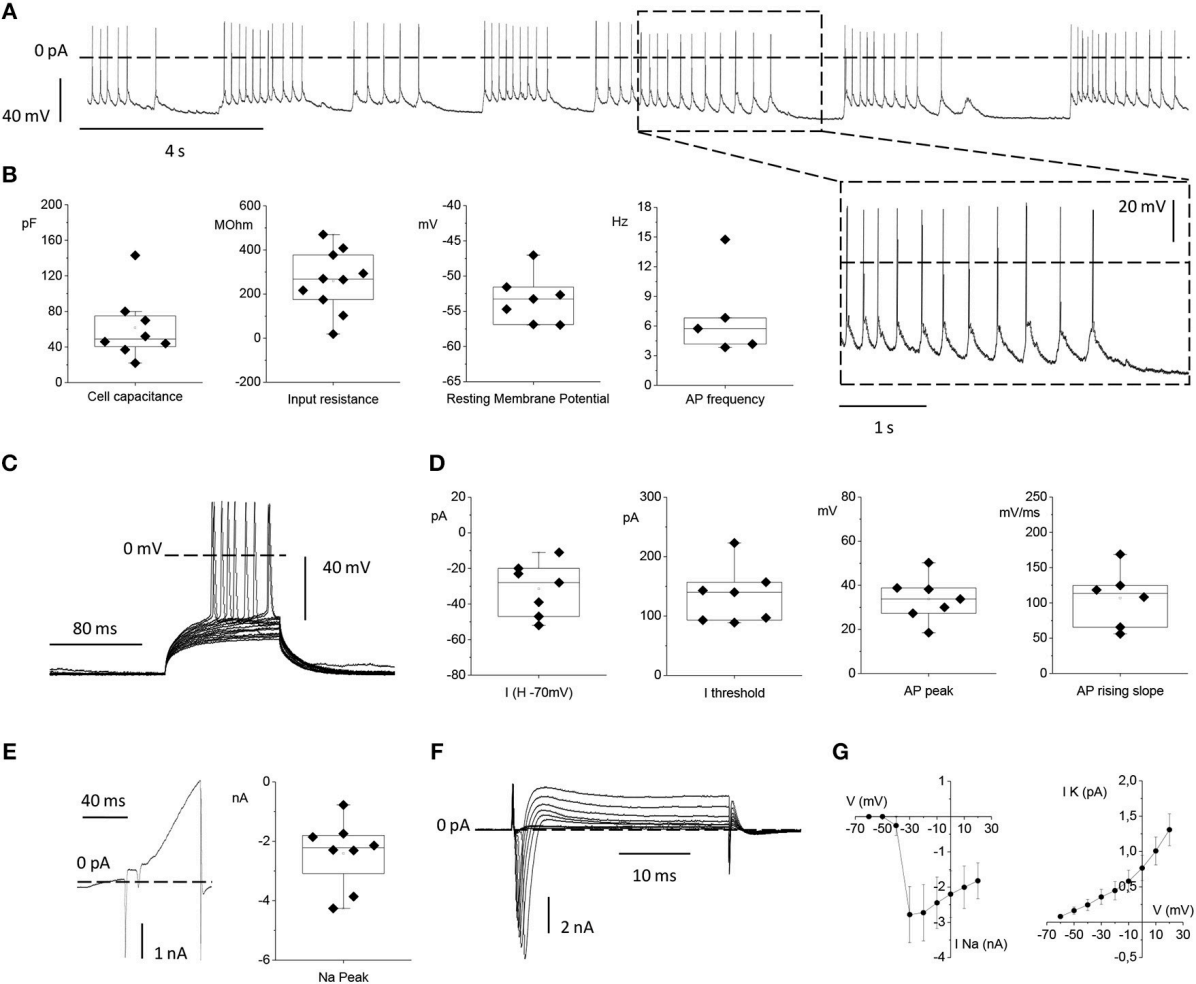
Electrophysiological properties of primary rat cortical neurons plated on top of the MTJ sensor. **(A)** Representative traces of spontaneous action potential (AP) firing recorded in current clamp configuration from a cortical neuron. Inset: an enlarged detail of the APs firing activity. **(B)** From left to right: Box plots of cell capacitance (*N* = 8), input resistance (*N* = 10), resting membrane potential (*N* = 7) and AP firing frequency (*N* = 5). **(C)** Evoked AP firing activity elicited by +5 pA increasing current steps from a Holding Potential (VH) of −70 mV. **(D)** From left to right: Current injected for maintaining the HP at −70 mV (*N* = 7), minimum current injected for eliciting AP activity from a HP of −70 mV (*N* = 7), AP peak (*N* = 7) and maximal AP rising slope (*N* = 6). **(E)** Whole-cell currents recorded in voltage-clamp configuration with a ramp protocol depolarizing the cell from −100 to 120 mV in 100 ms (left) and sodium current peak (right) (*N* = 8). **(F)** Inward sodium and outward potassium currents evoked by the depolarizing voltage steps from −70 to 20 mV in 10 mV increments. **(G)** Current vs. Voltage (IV) relationships relative to the mean peak sodium current (left) (*N* = 6) and the mean steady state potassium current (right) (*N* = 9). Data are from 3 independent preparations with neurons plated on 3 MTJ sensor chips.

To test the functionality of neurons grown on the top of MTJ sensors, we analyzed their electrophysiological properties by means of patch-clamp recordings. We plated primary cortical rat neurons on top of MTJ devices and recorded them from 14 to 21 days after plating. At this age, primary *in vitro* cortical neurons develop a sustained and persistent spontaneous AP firing activity that we measured in current-clamp configuration (Figure 7A).

The passive membrane properties, namely: the neuronal capacitance, which furnishes a rough estimation of the cell size (61.7 ± 13.3 pF), the Vrest that was −53.3 ± 1.3 mV and the input resistance (R_in_) that was 259.8 ± 43.9 MΩ (Figure 7B) were in full agreement with the corresponding values previously observed under standard culture conditions (Yang et al., 1996; Bean, 2007; Staiger et al., 2016). The spontaneous firing activity that neurons plated on the MTJ devices were able to generate at the resting potential showed a frequency of 7.07 ± 1.99 Hz (Figure 7B).

We also studied evoked AP firing (Figure 7C) in neurons maintained at a Vh of −70 mV through the injection of a negative current of −31.4 ± 5.7 pA. The mean current threshold necessary to elicit the first AP from a Vh of −70 mV (rheobase) was 134.6 ± 18.0 pA. APs evoked during depolarizing steps reached a maximum membrane voltage (AP peak) of 33.8 ± 3.8 mV with an average maximal rising slope of the upstroke phase of 107.01 ± 16.91 mV/ms (Figure 7D).

Lastly, we analyzed voltage-gated sodium and potassium currents underlying the generation of the firing activity under voltage-clamp conditions. A linear ramp stimulation protocol, from −100 to +120 mV, elicited a current response characterized by (i) a fast and transient inward (negative) peak generated by voltage-gated sodium channels, followed by (ii) a slower outward (positive) current activated at more depolarized potentials, generated by voltage-gated potassium channels (Figure 7E). The current peak amplitude of the initial negative peak (sodium peak) was −2.4 ± 0.4 nA (Figure 7E). Alternatively, voltage-gated sodium and potassium currents were elicited by depolarizing the neuron with increasing voltage steps, from −60 to +20 mV, (Figure 7F). Even in this case, faster and inactivating inward sodium currents appeared at the beginning of each voltage step, while slower outward potassium currents were recorded at steady state. Current vs. voltage (I/V) relationships were plotted with the mean sodium and potassium currents as a function of the clamped voltage steps (Figure 7G). The resulting voltage-dependence was consistent with data obtained from similar cortical neuronal preparations (Yang et al., 1996; Bean, 2007; Staiger et al., 2016). Indeed, neurons grown in contact with MTJ sensors displayed normal electrophysiological activity, and their spontaneous firing activity generated by the expression of sodium and potassium channels, testifies a correct physiological maturation of the intrinsic excitability properties. These results obtained with the sensitive electrophysiological approach confirm a correct development and maturation of neurons grown in contact with the MTJ sensors.

## Discussion

In this work, we show the biocompatibility of MTJs for *in vitro* neuron culture studies. We first demonstrated the preservation of the magnetic proprieties of the MTJ sensors after 31 days of permanence in culture medium. Moreover, we achieved fully viable on-chip neuronal networks for periods longer than 20 DIV and monitored the proper neuronal network growth and maturation under these conditions with immunofluorescence and patch-clamp studies. No constraints were found for primary neuronal culture growth on the MTJ chip surface. We also successfully grew neuronal networks with controlled topology through a micropatterning technique that likely promotes the best conditions to detect neuronal magnetic signals. In conclusion, this work establishes the biocompatibility of a MTJ chip for neuronal studies *in vitro*.

These results validate the possibility of using a magnetoresistive platform to detect biosignals originating from the spontaneous and evoked electrical activity of primary neurons in culture. Indeed, three main ingredients contribute to the sensitivity of a magnetic platform: the intensity of the magnetic signal in correspondence with the sensor surface, the sensitivity of the MTJ device and the sensitivity of the electronic acquisition platform. Regarding the first point, engineered cultures allow neurons to grow on top of the sensors with neurites aligned in a controlled topology. In this way, the magnetic field generated by the action potentials propagating in the axons can be aligned to the sensitive direction of the sensors. In addition, the thin capping layers optimized in this work allow reducing the distance between the neurons and the sensor surface. In this configuration, highly sensitive magnetic tunneling junctions with sensitivities in the range of low-nT/ pT can be employed to record the weak and rapidly decaying magnetic signals arising from the neuronal activity. Finally, to maintain the low noise levels required by the small signals expected, *ad hoc* acquisition platforms can be developed. Some of the authors designed and built a platform based on a generation channel to drive all the sensors with a sinusoidal voltage and four low-noise parallel acquisition channels (Sharma et al., 2017b). The front-end acquisition channels in combination with a Field Programmable Gate Array can process four channels simultaneously with input voltage noise of only 3 nV/√Hz.

Our results pave the way for a new generation of biomagnetic chips to study the neuronal magnetophysiology *in vitro* with high spatio-temporal resolution.

## Author Contributions

DP and FB conceived the study. DM, DP, and FB designed the experiments. PS, EA, MM, and DP fabricated and characterized the sensors. DM carried out neuronal culture experiments and analysis. SD supported the microprinting coating deposition experiments. MD contributed with electrophysiology experiments and analysis. DP, RB, PB, and FB gave conceptual advice and reviewed the manuscript. DM and DP prepared the manuscript. All co-authors agreed to the submission of the final manuscript.

### Conflict of Interest Statement

The authors declare that the research was conducted in the absence of any commercial or financial relationships that could be construed as a potential conflict of interest.

## Supplementary Material

Video S1 The video shows a patterned coating deposition on a top of a MTJ sensor by a nano-drop ink jet print (NanoEnabler, BioForce Nanoscience Inc.), equipped with a cantilever (NanoandMore). The cantilever reservoir contains 0.3 μl of the coating solution (PEI). After micrometric alignment of the chip in correspondence of the cantilever, the coating deposition starts in order to obtain PEI line exactly on top of the MJT sensor. This process allow to perform a controlled topology neuronal networks. Scale bar 20 μm. Video was edited using VSDC Free Video Editor, version 5.8.9.
